# Self-Targeting of Carbon Dots into the Cell Nucleus: Diverse Mechanisms of Toxicity in NIH/3T3 and L929 Cells

**DOI:** 10.3390/ijms22115608

**Published:** 2021-05-25

**Authors:** Markéta Havrdová, Iztok Urbančič, Kateřina Bartoň Tománková, Lukáš Malina, Janez Štrancar, Athanasios B. Bourlinos

**Affiliations:** 1 Regional Centre of Advanced Technologies and Materials, Czech Advanced Technology and Research Institute, Palacký University, Křížkovského 511/8, 779 00 Olomouc, Czech Republic; 2 Laboratory of Biophysics, Condensed Matter Physics Department, “Jozef Stefan” Institute, Jamova 39, 1000 Ljubljana, Slovenia; iztok.urbancic@ijs.si (I.U.); janez.strancar@ijs.si (J.Š.); 3 Department of Medical Biophysics, Faculty of Medicine and Dentistry, Institute of Translation Medicine, Palacký University in Olomouc, Hněvotínská 3, 775 15 Olomouc, Czech Republic; katerina.barton@upol.cz (K.B.T.); lukas.malina@upol.cz (L.M.); 4 Physics Department, University of Ioannina, 45110 Ioannina, Greece; bourlino@cc.uoi.gr

**Keywords:** carbon dots, fluorescence microspectroscopy, cnucleus, nucleolus, cytotoxicity, genotoxicity, fibroblasts, NIH/3T3, L929

## Abstract

It is important to understand the nanomaterials intracellular trafficking and distribution and investigate their targeting into the nuclear area in the living cells. In our previous study, we firstly observed penetration of nonmodified positively charged carbon dots decorated with quaternary ammonium groups (QCDs) into the nucleus of mouse NIH/3T3 fibroblasts. Thus, in this work, we focused on deeper study of QCDs distribution inside two healthy mouse NIH/3T3 and L929 cell lines by fluorescence microspectroscopy and performed a comprehensive cytotoxic and DNA damage measurements. Real-time penetration of QCDs across the plasma cell membrane was recorded, concentration dependent uptake was determined and endocytic pathways were characterized. We found out that the QCDs concentration of 200 µg/mL is close to saturation and subsequently, NIH/3T3 had a different cell cycle profile, however, no significant changes in viability (not even in the case with QCDs in the nuclei) and DNA damage. In the case of L929, the presence of QCDs in the nucleus evoked a cellular death. Intranuclear environment of NIH/3T3 cells affected fluorescent properties of QCDs and evoked fluorescence blue shifts. Studying the intracellular interactions with CDs is essential for development of future applications such as DNA sensing, because CDs as DNA probes have not yet been developed.

## 1. Introduction

Intracellular labeling of cells by nanomaterials is widely used in many “nano-bio” studies. Thus, advanced information on carbon dots (CDs) inside the nucleus is very important for understanding the nanoparticle’s trafficking mechanisms. CDs have color-tunable and stable fluorescent properties, high biocompatibility, low cytotoxicity and excellent cell membrane permeability [1,2,3,4]. For these advantages, CDs demonstrate many application-promising features, providing their exploitation in a wide spectrum of fields, such as chemical sensing [5,6], biosensing [7], bioimaging [8], catalysis [9,10,11], light-emitting diodes [12] and solar cells [13,14]. Compared to common quantum dots and organic dyes, photoluminescent CDs are superior in terms of high aqueous solubility, environment friendly composition, easy functionalization, high resistance to photobleaching, low toxicity and good biocompatibility [4,15]. These unique characteristics have allowed to employ CDs for bioassays [16], photothermal therapy [17,18], nanomedicine [19,20,21], with a good potential also in clinic therapy [22], especially for detection of various type of diseases such as neurodegenerative disorders (Alzheimer’s (AD), Parkinson’s (PD), Huntington’s) and systemic lysozyme amyloidosis [23] or cancer [24,25,26,27]. Nowadays, many different methods of CD fabrication are known [28]: laser ablation [29], acidic/thermal oxidation [30], electrochemical synthesis [31], hydrothermal treatment [32] and microwave irradiation [33]. Moreover, green preparation processes using natural sources have also been adopted [34,35,36,37]. CD synthesis has thus seen a remarkable progress, but selective targeting of cellular structures or specific cell types has remained a challenge for widespread applications of CDs in living cell imaging and tracking. Despite the fact that effective and common strategy to enable the entrance of CDs into the nucleus is to functionalize their surface by substances targeting the organelles [38,39], we first presented nonmodified CDs inside the cell nucleus in 2014 [40]. Generally, the most reported information on subcellular distribution of CDs describes interactions with cytoplasm [41,42] and organelles such as mitochondria [43,44], Golgi apparatus [45,46] and lysosomes [47]. The presence of bare CDs in the nuclear localization is very rare [1] because a nuclear envelope protects genetic material from chemical reactions that are occurring elsewhere in the cell. The intranuclear environment is surrounded by double phospholipid membrane which has an outer and inner part and contains nuclear pore complexes (NPCs). The main task of NPCs is to provide a communication pathway between cytosol and nucleus [48,49]. Penetration of nanoparticles into the nucleus is limited by the diameter of nuclear pores, which are protein complexes composed of nucleoporins intersecting the nuclear envelope [50]. Number of NPCs embedded into the nuclear membrane of one human eukaryotic cell is 3000–4000 [51]. The size and structure of the NPCs varies between individual types of eukaryotic cells (from yeast to higher eukaryotes) or can be species-specific (vertebrates vs. invertebrates) [52]. For example, oocytes of Xenopus have a length of the central pore of ~90 nm and a diameter in the narrowest place (in the middle part of the NPC) of 45–50 nm. The widest part of NPC is on the nuclear periphery and has a diameter of ~70 nm [53,54]. Molecules enter the nucleus through two mechanisms according to their size. Small molecules and proteins with size of less than 50 kDa penetrate across the nuclear membrane in both directions (from cytosol to the nucleus, from the nucleus into cytosol) in a passive way (diffusion) using the water channels which have a diameter of ~9 nm in NPC [55,56]. Consequently, nanoparticles bigger than 9 nm cannot enter in the nucleus by the mentioned mechanism [57]. For example, HeLa cancer cells have thinner water channels, therefore, penetration through them is more limited by the size of nanoparticles. According to the study [58], only proteins with a diameter up to 2.5 nm are able to cross the channels by diffusion. Another study mentioned that the size of transported molecules by diffusion is 4.9–5.7 nm [59]. However, larger macromolecules (proteins and RNA) cannot go through the open channels. For this reason, bigger proteins use an active mechanism based on a selective transport in one way (from cytosol into the nucleus or from the nucleus in cytosol). These signal channels can open up to diameters of around 25 nm [55]. Unfortunately, detailed information on the NPC diameter of L929 and NIH/3T3 cell lines was not reported yet. Nevertheless, it is generally known that only nanoparticles with a diameter smaller than 30 nm might be imported through nuclear pores [60].

In this work, we used fluorescent microspectroscopy (FMS, spectral imaging) to obtain detailed knowledge about the cellular uptake and biodistribution of positively charged CDs inside cells, especially in the nucleus and nucleolus. Positive nanoparticles are able to disrupt endosomes via the “proton sponge” effect, enter to cytosol and progress to the nucleus where they can interact with DNA [61,62,63]. The NIH/3T3 mouse embryonic cell line is the standard cell line of the fibroblasts, which have a pivotal role with their versatile functions in the body [64]. Thus, we also determined CD cytotoxicity and genotoxicity in NIH/3T3 and compared these results with commonly used mouse L929 fibroblasts [65,66] to assess a different sensitivity of individual cell lines to the strongly positively charged CDs.

## 2. Results

### 2.1. Detection of QCDs Inside Nuclei, Viability and DNA Damage

Cellular uptake and bio-distribution of QCDs were observed by fluorescence microspectroscopy (FMS), which provides information on the local molecular surroundings (spectral shifts due to local changes) at a microscopic spatial resolution. Both cell lines (NIH/3T3, L929) were incubated with various doses of QCDs, 50, 100, 200 and 400 µg/mL, for 24 h and imaged. To obtain better information on the interaction of cells with a nanomaterial, live monitoring was done immediately after addition of QCDs (400 µg/mL) to see main changes in the highest concentration. QCDs target the nuclei of all the cells very intensively, thus, microscopic experiments were supported by measurements of viability and DNA damage.

#### 2.1.1. Results of QCD-Labeled NIH/3T3 Cells

**Figure 1:**
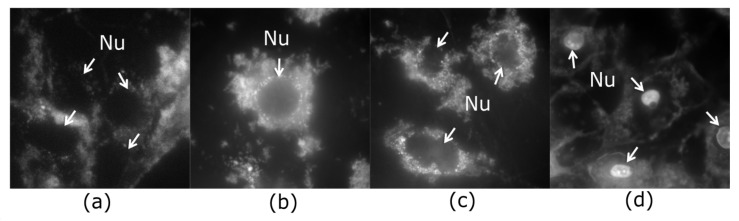
Fluorescent imaging of NIH/3T3 cells exposed to QCDs for 24 h: (**a**) 50 µg/mL; (**b**) 100 µg/mL; (**c**) 200 µg/mL; (**d**) 400 µg/mL; “Nu” and arrows denotes the nuclei. FMS, field of view 91 µm.

**Figure 2:**
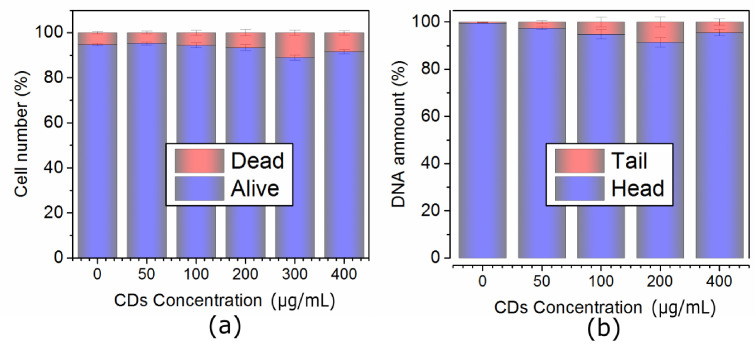
(**a**) Viability and (**b**) genotoxicity of NIH/3T3 cells exposed to the concentration line of QCDs and incubated for 24 h. Please note the following genotoxicity terms: “head” is intact DNA in nucleus, “tail” is damaged DNA migrated away from the nucleus; when the tail value is more than 10%, the dose is genotoxic.

In NIH/3T3 cells, QCDs were incorporated into cytosol from the concentration of 50 µg/mL and accumulated in perinuclear area up to 200 µg/mL (Figure 1a–c). The first sign of carbon dots inside the nucleus occurred at 300 µg/mL, subsequently, all NIH/3T3 cells had filled nuclei and nucleoli after exposure to 400 µg/mL (see Figure 1d). These results were also confirmed by live monitoring of QCD-treated NIH/3T3 cells, when the labeling of nuclear membrane started after 50 min from cell incubation with a QCDs concentration of 400 µg/mL (see Video S1). Interestingly, viability of NIH/3T3 cells was not changed even after exposure to the highest concentration either (Figure 2a). Moreover, the results from comet assay in genotoxicity testing showed that QCDs at concentrations of 50–400 µg/mL did not evoke notable DNA damage (Figure 2b).

**Figure 3:**
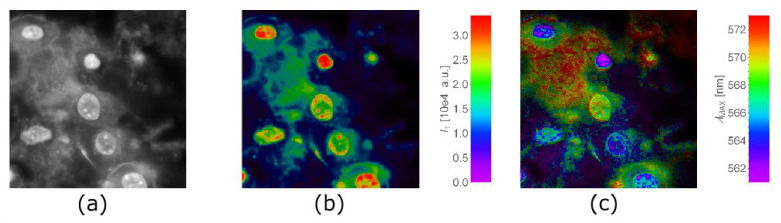
The nuclei of NIH/3T3 cells filled with QCDs. (**a**) Fluorescence intensity image generated by summing up all the images in the λ-stack (FMS, field of view 132 µm); (**b**) False colored maps of intensity; (**c**) Spectral peak position obtained from spectral fitting. Concentration of QCDs was 400 µg/mL with an incubation time of 24 h.

Due to the nontoxic behavior of QCDs, detailed observation of intranuclear changes was conducted. This cell line opened options to knowing more about incorporated carbon dots in the nucleus, thus, we focused on detailed microspectroscopy of intranuclear QCDs (Figure 3). NIH/3T3 cells were incubated for 24 h with a concentration of 400 µg/mL. Subsequently, QCD-labeled cells were washed by PBS and observed in an HEPES solution using FMS (Figure 3a). From fluorescence intensity, it was obvious that QCDs concentrated in the nuclei and nucleoli. Nevertheless, each cell has a different size and metabolic activity, resulting also in varying amount of nanomaterial inside nuclei (see false-colored Figure 3b). The lowest intensity of QCDs was observed in cytosol (green-stained places), the highest occurrence of QCDs was found in the nuclei and nucleoli (red color).

If we compare these results of fluorescence intensity with changes in emission wavelength of QCDs inside cells, the highest intensity (red areas in Figure 3b) corresponds to the intracellular places with the shortest emission wavelength (violet areas in Figure 3c). Particles outside the cell had the longest emission wavelength (in our presentation red color in the Figure 3c).

**Figure 4:**
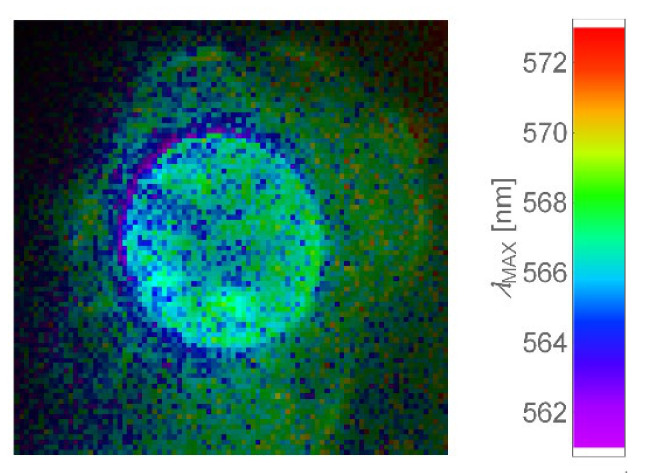
Detailed image of the nucleus filled with QCDs (400 µg/mL, 24 h, washed in PBS) shown in blue color and nucleus membrane represented by a violet ring. NIH/3T3 cells; field of view of 55 µm.

One possible reason of the mentioned spectral shifts could be ascribed to differences in sizes of QCDs. The more particles in the cell, the more they are exposed to defense mechanisms which may decrease their size in the interest to protect the cell. Thus, “violet” nuclear envelope (Figure 4) can be caused by a strong protective mechanism promoting to split the nanomaterial and save the cell, because the cellular defense begins shortly after endocytosis, whereby in endo/lysosomes there is acidic pH [67]. Here, it should be stressed that cytosol is full of self-defense mechanisms to protect cells against microbial DNA, RNA and other pathogens [68]. Moreover, as we mentioned before, penetration of nanoparticles into the nucleus is limited by the sizes of nuclear pores [52,53,54,55,56,57,58,59,60]; previous studies [55,56] have already described penetration of proteins across the nuclear membrane in a passive way (diffusion) using the water channels which have an NPC diameter of ~9 nm. The NPC size of NIH/3T3 and L929 cells has not been studied yet. Thus, it could happen that through the nuclear pores penetrated just the smallest QCDs from the whole fraction (QCDs 4–9 nm); here, the main size of QCDs is 7 nm and could have size-dependent fluorescence behavior—shorter emission wavelength for smaller QCDs—or only the individual QCDs reached the nucleus. Different wavelength could be also caused by interactions of QCDs with intracellular molecules and compartments. Nevertheless, carbon dots also interact between themselves. We found that a red shift of emission wavelength occurred with increasing concentration of QCDs [69], similar to organic dyes upon aggregation [70]. This phenomenon is obvious in the case of QCDs around cells and on the surface of the cellular membrane (Figure 3c). It is probably caused by agglomeration of nanomaterial evoked by CD–CD interactions. However, after incorporation of the nanomaterial into the cells, emission wavelength was found to be blue-shifted in the nucleus compared to the cytosol, which could arise from breaking the QCD aggregates of size-filtering of QCDs while passing NPCs.

#### 2.1.2. Results of QCD-Labeled L929 Cells

**Figure 5:**
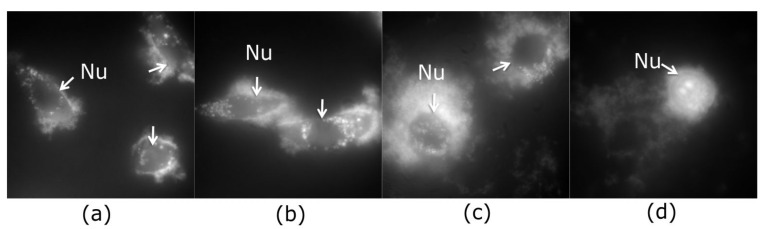
Fluorescent imaging of L929 cells exposed to QCDs for 24 h: (**a**) 50 µg/mL; (**b**) 100 µg/mL; (**c**) 200 µg/mL; (**d**) 400 µg/mL—dead cell with QCDs inside the nucleus and nucleoli; “Nu” and arrows denotes the nuclei. FMS, field of view 91 µm.

**Figure 6:**
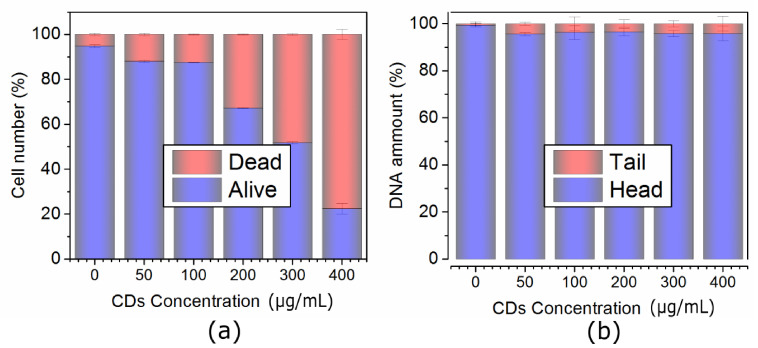
(**a**) Viability and (**b**) genotoxicity of L929 cells exposed to the concentration line of QCDs and incubated 24 h. Please note the following genotoxicity terms: “head” is intact DNA in nucleus, “tail” is damaged DNA migrated away from the nucleus; when the tail value is more than 10%, the dose is genotoxic.

Second L929 cell line interacted with the same doses of QCDs like NIH/3T3 cells and were also incubated for 24 h. Concentrations of 50–100 µg/mL did not cause any significant changes (Figure 5a,b), however, 200 µg/mL altered cellular morphology into a ring shape (Figure 5c). From the microscopic images, it was obvious that carbon dots accumulated in the endo/lysosomes in the perinuclear area. Higher concentrations, i.e., 300–400 µg/mL, caused a weak cellular adhesion followed by cellular death (Figure 5d and Figure 6a). From the live monitoring, it was also obvious that QCDs (400 µg/mL) entered into the cells after 40 min and evoked blebbing, which is the first sign of cell death (see Video S2). Moreover, just very few cells persisted on the bottom of the dish after 24 h. The rest of the cells had full nuclei and nucleoli of QCDs and were slightly floating (Figure 5d). These results are also confirmed by the measurements of viability, see Figure 6a. QCDs did not induce any DNA damage (Figure 6b) to this cell line, probably due to the occurrence of many dead cells in the higher concentrations and fast cellular dead. QCDs did not target the nucleus in living L929 cells, thus, safety dose of QCDs for this cell line was assigned to concentrations of up to 100 µg/mL.

### 2.2. Concentration Dependent Uptake and Endocytosis of Both Cell Lines

**Figure 7:**
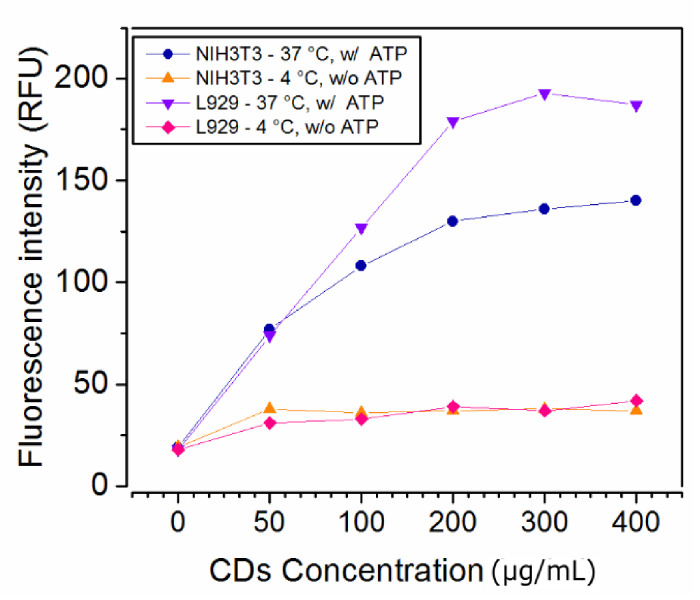
Concentration dependent uptake and endocytosis of both cell lines: blue and violet lines—NIH/3T3, L929 cells incubated in standard conditions; orange and pink lines—NIH/3T3, L929 cells labeled after inhibition of endocytosis.

The microscopic results discussed above showed that the uptake of QCDs by individual cell lines is distinct. The mechanism of QCDs incorporation into cells generally depends on many distinct factors: charge and size of carbon dots, cellular type and their ability to proliferate in the presence of a nanomaterial [1,2,3,4,5,6,61]. Nevertheless, the main mechanism of uptake is endocytosis [71]; thus, we compared the fluorescence intensity of labeled cells incubated at standard conditions (37 °C and standard levels of ATP) with the fluorescence intensity of cells incubated at conditions, where endocytosis is inhibited (4 °C and depleted levels of ATP). NIH/3T3 and L929 cells were incubated with QCDs at concentrations of 0–400 µg/mL for 24 h at 37 °C and, in parallel, the cells with inhibited endocytoses labeled with the same concentration line were incubated for 24 h at 4 °C, subsequently trypsinized and measured by the flow cytometer (Figure 7). From the observed results at 37 °C (see blue and violet line on the Figure 7), it is obvious that the uptake of both cell lines is similar up to a concentration of 50 µg/mL. The main changes occurred at a dose of 100 µg/mL, when the uptake of L929 cells rapidly began to be faster than that of NIH/3T3 cells. These results show that L929 cells have the strongest uptake at a concentration range of 50–200 µg/mL; at higher concentrations, the changes are not so significant, probably because of cell death. The uptake of NIH/3T3 cells gradually grew with increasing concentration and persisted up to the highest doses. In both cell lines, it seems that the concentration of 200 µg/mL is close to saturation. In the case of cells with inhibited endocytosis (see orange and pink line in Figure 7), we confirmed that the uptake of medium with QCDs is minimal. Therefore, for the uptake of QCDs, both cell lines used energy dependent uptake—endocytosis.

**Figure 8:**
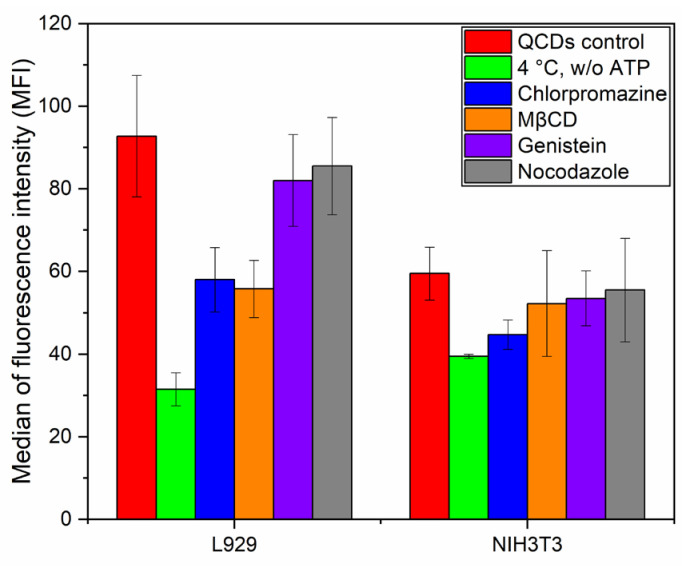
Inhibition of different endocytic mechanisms in L929 and NIH/3T3 cells immediately after adding the QCDs concentration of 200 µg/mL; incubation time of inhibitors was 2 h.

Cells can generally use different processes to internalize extracellular materials and several mechanisms of endocytosis have been identified [72]. The best-characterized endocytic pathway is clathrin-mediated endocytosis [73], which was also described as the main pathway for the uptake of nanoparticles smaller than 200 nm [74]. Another form of endocytosis is clathrin-independent endocytosis linked to the caveolae system, mediating the process of so-called potocytosis. The main target of the material internalized by this type of endocytosis is not the endo/lysosome but the endoplasmic reticulum and Golgi apparatus. This process is based on the caveoles (“holes”) of the cell membrane, which contain the caveolin protein and form the caveolar coat [72]. Both type of endocytosis (clathrin-dependent/-independent) are selective for certain groups of ligands. However, a nonspecific endocytosis also exists in all mammalian cells—pinocytosis (fluid phase endocytosis) [67], that is defined as the actin-driven nonspecific bulk uptake of extracellular fluid during which the vesicles are greater than 2 µm [74]. In our results, we confirmed internalization of QCDs by endocytosis in both cell lines; nevertheless, uptake was significantly different. Thus, we used transport inhibitors to obtain information on the type of endocytosis of cells labeled by 200 µg/mL of QCDs. Chlorpromazine was used to block clathrin-mediated endocytosis and macropinocytosis. Methyl-β-cyclodextrin (MβCD) is a common agent for cholesterol depletion and affects not only caveolae-mediated endocytosis, but almost every endocytic mechanism (strongly depending on the concentration used and cell type tested) [75,76]. Genistein is a tyrosine kinase inhibitor for caveolae-mediated endocytosis [77]. Nocodazole is a compound to block microtubule polymerization and microtubules are essential components for clathrin-mediated endocytosis [78], macropinocytosis [79,80], and they also affect mitosis [81]. The results show (Figure 8) that L929 cells had a significant decrease of uptake after inhibition of clathrin-mediated endocytosis and macropinocytosis by chlorpromazine. The same results were also observed after inhibition of pathways for uptake through the lipid rafts and caveolae-mediated endocytosis by MβCD. On the contrary, inhibition by genistein (inhibitor of caveolae-mediated endocytosis) and nocodazole (binds to tubulin and block microtubule polymerization) did not affect uptake of nanoparticles so much. L929 cells probably internalize QCDs by caveolae-mediated endocytosis because inhibition of caveolae-mediated endocytosis by genistein did not cause interruption of QCD internalization. Moreover, the uptake was also significantly reduced after cholesterol depletion by MβCD, meaning inhibition of pathways for lipid rafts, caveolae-mediated endocytosis. Internalization of QCDs after nocodazole inhibition was not reduced; nevertheless, it can be caused by the very small size of carbon dots—inhibition of cellular uptake has been described mainly in larger particles (~200 nm) [82]. If we compare these results with the study on L929 cells labeled by small transfection complexes of PEI/DNA [83], we can confirm similar pathways and barriers for transfection. QCDs after adhesion to the cell membrane were endocytosed. In lower doses (up to 200 µg/mL), endo/lysosomes persisted in the perinuclear area after 24 h. However, at a high concentration (400 µg/mL), a lytic process in endo/lysosomes probably released QCDs into the cytoplasm (see Video S2), which were transported by nondefined mechanism to the nucleus and entered into nuclei and nucleoli in a short time without the need to enter mitosis. It seems that a high concentration of QCDs disrupted nuclear membrane, penetrated to the nucleus, and evoked cellular death. From these reasons, strongly positively charged QCDs are not suitable for labeling of nuclei of L929 cells. On the contrary, the second NIH/3T3 cell line showed a different behavior. From all the results presented in this study, it is obvious that the uptake of QCDs by NIH/3T3 cells is slower and more prudent. After 2 h inhibition of different types of endocytosis, we could see only mild reduction of uptake. The greatest decrease occurred in clathrin-mediated endocytosis inhibited by chlorpromazine; the values of other inhibitions were comparable in terms of error bars with control untreated cells. Thus, we cannot exactly state which type of endocytosis was used by NIH/3T3 cells to internalize QCDs. Nevertheless, in the study in [84], the authors described the uptake of Au nanoparticles (~20 nm) by HeLa cells undergoing clathrin-mediated endocytosis.

Cellular damage depends on the ability of repairing DNA in the cell cycle, where only cells with intact DNA can continue to mitosis and cells with damaged DNA undergo cellular death [85]. Thus, the cell cycle profile was measured.

### 2.3. Cell Cycle Analysis of Both Lines

**Figure 9:**
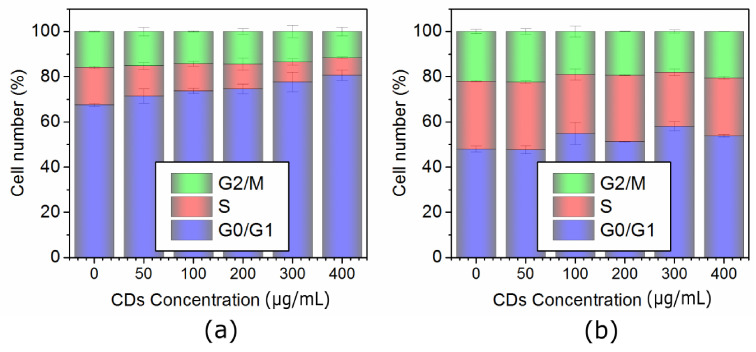
Cell cycle profile of cells labeled with QCDs: (**a**) Healthy mouse NIH/3T3 fibroblasts, (**b**) Healthy mouse L929 fibroblasts.

The cell cycle profile was analyzed by the flow cytometer mentioned above using DNA kit (BD CycletestTM Plus DNA kit). The results showed that the cell cycle of NIH/3T3-labeled cells had an arrested G0/G1 phase and shorter S phase (see Figure 9a). These abnormalities were more pronounced with the increasing concentration of QCDs. DNA repair can be responsible for G0/G1 arrested phase, because, from the results of genotoxicity, it is obvious that DNA was not damaged (Figure 2b) and the percentage of viability was also high (Figure 2a). The second hypothesis suggests that the observed results could be a sign of inhibition of cell proliferation observed with increasing doses of QCDs. Regardless, NIH/3T3 cells could slow down proliferation, visible in higher concentration of the sample, because the cells are overloaded by the particles and have limited movement. The cell cycle profile of QCD-treated L929 cells is not so different from control cells (Figure 9b) and did not have a prolonged G0/G1 phase in which DNA is repaired unlike NIH/3T3 cells. It seems that L929 cells probably did not repair DNA damages which were not present also in the genotoxicity measurements (Figure 6b), as the sample evoked cell death very fast (Figure 6a).

## 3. Materials and Methods

### 3.1. Carbon Dots

Quaternized carbon dots (QCDs) were prepared by thermal oxidation of a tris(hydroxymethyl)aminomethane (Tris)—betaine hydrochloride salt precursor, where Tris provides the carbon source and betaine the surface modifier [86]. The surface of the dots is decorated with pending quaternary ammonium groups (i.e., −N(CH_3_)_3_^+^) which confer positive surface charge and high aqueous dispersibility. QCDs show a uniform size/surface charge distribution with an average particle size of 7 nm and quasi-spherical morphology. For more information on the material characteristics, see our previous studies [86,87]. It should be noted that QCDs were synthesized at 250 °C and displayed a normal for carbon dots quantum yield of 4%. Hence, the formation of low molecular weight fluorophores with much brighter fluorescence should be rather excluded, since such a formation is usually favored at temperatures <200 °C [88,89]. Furthermore, the purity of the dots was evidenced by capillary electrophoresis, showing a single narrow peak in the corresponding chromatograph of QCDs [86].

### 3.2. Cell Cultivation

Both cell lines (NIH/3T3, L929) were purchased from American-type Culture Collection (ATCC Washington, DC, USA). Mouse NIH/3T3 fibroblasts cells were cultivated in low glucose Dulbecco’s Modified Eagle medium (DMEM, Life Technologies, Carlsbad, CA, USA). Mouse L929 fibroblasts cells were cultivated in high glucose DMEM (Life Technologies). Both media contained also 10% fetal calf serum (FSC) and 10,000 U/mL penicillin and 10,000 µg/mL streptomycin. Cells were incubated under a special condition at 37 °C and under a 5% CO_2_ enriched atmosphere.

### 3.3. Fluorescence Microspectroscopy

**Figure 10:**
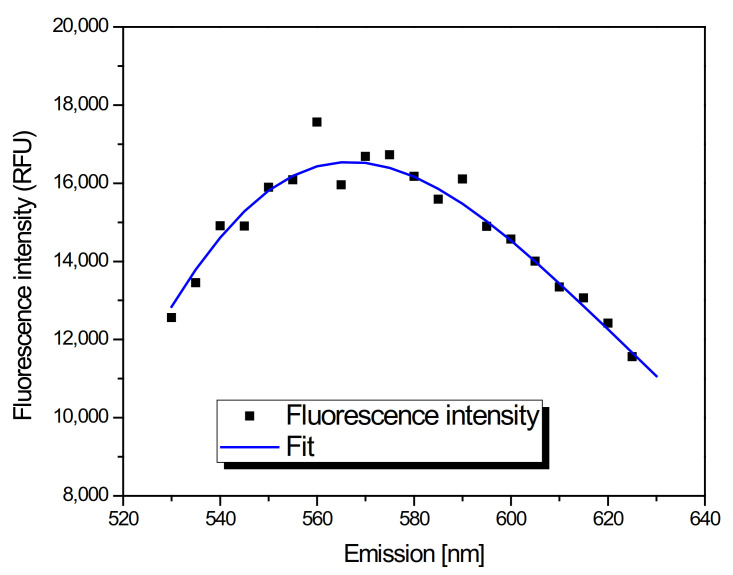
Spectral fitting of local intensities and emission spectra maximum wavelengths.

FMS is the technique based on a white light spinning disk inverted microscope with emission wavelength selection by a liquid crystal tunable filter (Varispec VIS-10-20, CRi, MA, USA) that provides spatially resolved detection of local molecular environment [90]. In brief, images at different wavelengths are acquired sequentially through a 100×/1.4 oil or 60×/1.27 waterimmersion objective (Nikon, Tokyo, Japan) using an EMCCD camera (Andor iXon3 897, Belfast, UK), from which emission spectra in every pixel are extracted. Local intensities and emission spectra maximum wavelengths were obtained using custom spectral fitting software written in Wolfram Mathematica [91], as demonstrated in Figure 10. Since CDs can be excited at different wavelengths [92,93], we first measured the fluorescent signals of the QCDs in solution with various available excitation/emission filters and chose the combination with the highest signal (excitation of 430–490 nm, emission of 506–594 nm, data not shown). To observe interaction of QCDs with cells, 10 × 103 of NIH/3T3 and L929 cells were seeded separately on glass-bottom cell culture dishes (standard 35 mm dish with the 12 mm glass viewing area, NuncTM, ThermoFisher Scientific, Waltham, MA, USA) and cultivated for 24 h at 37 °C and 5% CO_2_-enriched atmosphere. On the next day, QCDs were diluted into 200 µL of medium in each culture dish (just into the glass bottom area) to achieve different final concentrations (50, 100, 200, 400 µg/mL) and incubated for 24 h. Subsequently, the medium was removed and the culture dishes with cells were washed twice with PBS. After these steps, a solution of HEPES and PBS (1:9) was added into each dish and observed by FMS within 530–630 nm at 5 nm steps. For live monitoring of the uptake, QCDs (400 µg/mL) were added into the medium in which cells were grown and immediately observed for 2 h. The images and spectra were taken every 5 min, from which movies were generated in the Wolfram Mathematica program (see Videos S1 and S2).

### 3.4. Cell Cycle, Concentration Dependent Uptake, Endocytosis

Cell cycle, concentration dependent uptake, and endocytosis analysis were performed on a BD FACSVerse flow cytometer (BD biosciences, San Jose, CA, USA). DNA kit (BD CycletestTM Plus DNA kit, Becton Dickinson, Franklin Lakes, NJ, USA) was used according to the BD protocol after 24 h of cell incubation with various concentrations of QCDs. The fluorescent intensity of propidium iodide (PI) was measured using exc. 488 nm/em. 586 nm detector to determine any changes in the phases of the cell cycle.

Concentration-dependent uptake was evaluated based on the mean fluorescent intensity (MFI) of the labeled cells. We used concentrations of 50, 100, 200, 300 and 400 µg/mL of QCDs and after 24 h incubation, the supernatant was removed, and the cells were gently washed with PBS solution (0.1 M, pH 7.4). Then, cells were detached with trypsin (0.25% in EDTA, Sigma-Aldrich, St. Louis, MO, USA), resuspended in 100 μL of medium, and fluorescence intensity of labeled cells was analyzed on flow cytometer.

To determine whether the main mechanism of uptake is endocytosis, we compared the fluorescence intensity of labeled cells incubated under standard conditions (37 °C and standard levels of ATP) with the fluorescence intensity of cells incubated at conditions where endocytosis is inhibited (4 °C and depleted levels of ATP). Briefly, we put one plate with cells treated with different concentrations of CDs in the incubator with a temperature of 37 °C. The second plate with cells was treated with 10 mM 2-deoxy-d-glucose and 20 µM sodium azide for 1 h to cause ATP depletion. Then, we treated cells with different concentrations of QCDs and put the plate in conditions with a temperature of 4 °C. After 4 h of incubation, cells in both plates were trypsinized and the fluorescence intensity was measured on the flow cytometer.

Cellular uptake pathways were studied by different uptake inhibitors in order to determine the endocytosis mechanism. NIH3T3 and L929 cells were seeded in 96-well plate (10 × 103 cells/well). After 24 h, we treated cells with different inhibitors: 7.5 µg/mL of chlorpromazine (clathrin-mediated endocytosis inhibition), 2 mM of methyl-β-cyklodextran (depletion of cholesterol from cell membrane), 100 µM of genistein (caveolae-mediated endocytosis inhibition), and 20 µM of nocodazole (microtubule disruption) for 2 h. Then, cells were treated with 200 µg/mL of QCDs and incubated for 2 h more. At this step, control sample, not treated with any inhibitor was introduced and treated with 200 µg/mL of QCDs, as well. After incubation, supernatants were discarded, cells were collected, and median fluorescence intensity values were acquired using red fluorescent channel (ex. 488 nm/em. 586) on the flow cytometer. The sample with complete endocytosis inhibition was treated with 10 mM 2-deoxy-d-glucose and 20 µM sodium azide and incubated for 1 h, after QCDs treatment incubated in 4 °C for 2 h, and analyzed in the same way as the rest of the samples.

### 3.5. Viability

Viability of cells labeled with QCDs was determined by BD FACSVerse flow cytometer (BD Biosciences, USA) using LIVE/DEAD^®^ Viability/Cytotoxicity Kit (Thermofisher). Both cell lines were treated with the concentration line of 0–400 µg/mL of QCDs and incubated 24 h. Afterwards, cells were washed in PBS solution (0.1 M, pH 7.4), detached with trypsin (0.25% in EDTA, Sigma-Aldrich, St. Louis, MO, USA) and resuspended in 300 μL of medium. LIVE/DEAD^®^ Viability/Cytotoxicity Kit is the assay utilized to quantitate apoptotic cell death. Consequently, cells were incubated with 2 µL of ethidium bromide (2 mM) a 2 µL of calcein-AM (50 µM), diluted in DMSO. The fluorescence signal was measured on flow cytometry (red—exc. 488/em.700, green—exc.488/em.527). Red signal of ethidium bromide revealed dead cells which lost the membrane integrity while green cells had active intracellular esterases and catalyzed the nonfluorescent calcein-AM to highly fluorescent green calcein.

### 3.6. Comet Assay

Genotoxicity was determined by comet assay which detects DNA damage. This method is based on single cell gel electrophoresis (SCGE), whereby intact DNA stay in the nucleus (called “head”) and damaged DNA migrate away from the nucleus (resemble “tail” of comet). After staining by fluorescent probe specific for DNA, the image analysis compares fluorescent intensity of the nucleoid (head) and migrated DNA (tail) [94]. We adopted the methods from the study [95]. Microscope slides were precoated with 1% HMP agarose. After that, the cells were trypsinized, washed with DMEM with 10% FBS, and centrifuged (6 min, 1000 rpm). An amount of 85 μL of 1% LMP agarose was added to the cell suspension and 85 μL of this mixture was added to the microscope with agarose gel. The microscope slides were immersed in a lysis buffer for 1 h, and then placed in an electrophoretic tank and dipped into a cool electrophoresis solution for 40 min. Electrophoresis was run at 0.8 V/cm and 380 mA for 20 min. Finally, slides were neutralized in buffer (0.4 M Tris, pH = 7.5) and the samples were stained with SYBR^®^ Green and immediately scored using SW Comet Score (Sigma-Aldrich, St. Louis, MO, USA).

## 4. Conclusions

In this comprehensive study, we confirmed entering of positively charged QCDs with the average size of 7 nm into nuclei of both fibroblast cell lines, and described how different concentrations of the sample affect uptake (including endocytosis), cellular morphology, cytotoxicity, genotoxicity and cell cycle (. Each cell line interacted in a different way—NIH/3T3 were alive with QCDs in the nuclei/nucleoli, occurred from a concentration of 300 µg/mL, and were able to live with QCDs in nuclei/nucleoli as high as 400 µg/mL for 24 h. Moreover, very interesting shifting of emission wavelengths was captured in nuclear area of these labeled cells. In this case, no significant changes in the viability and genotoxicity occurred; only the cell cycle profile of labeled cells showed that the NIH/3T3 cells had probably undergone reparation of DNA. Uptake of QCDs in L929 nuclei/nucleoli was monitored from a concentration of 400 µg/mL, however, it led to apoptosis immediately. For 24 h exposure, the safety dose of 100 µg/mL was established by the measurement of viability; toxic concentration was observed at 300 µg/mL and no genotoxicity or cell cycle abnormalities were found. Results from the concentration-dependent uptake showed that L929 cells incorporated a significantly greater amount of QCDs after 24 h by endocytosis than NIH/3T3 cells. Most probably, this was the main reason of their rapid dying. The high permeability of CDs to the nuclei and nucleoli promises new approaches to DNA sensing and biological treatments, as CDs as DNA probes have not been developed yet.

## Supplementary Materials

The following are available online at https://www.mdpi.com/article/10.3390/ijms22115608/s1, Video S1: Real-time penetration of QCDs (400 µg/mL) into NIH/3T3 cells, Video S2: Real-time penetration of QCDs (400 µg/mL) into L929 cells.
